# Supplementary material for: Co/SiO2 Catalyst for Methoxycarbonylation of Acetylene: On Catalytic Performance and Active Species
Source: Molecules. 2024 Apr 26;29(9):1987. doi: 10.3390/molecules29091987 (PMC11085306; doi:10.3390/molecules29091987)
Supplement: Supplementary file 1 [file molecules-29-01987-s001.zip › molecules-2940411-supplementary.pdf]

# Supplementary Information

## Figures S1–S4

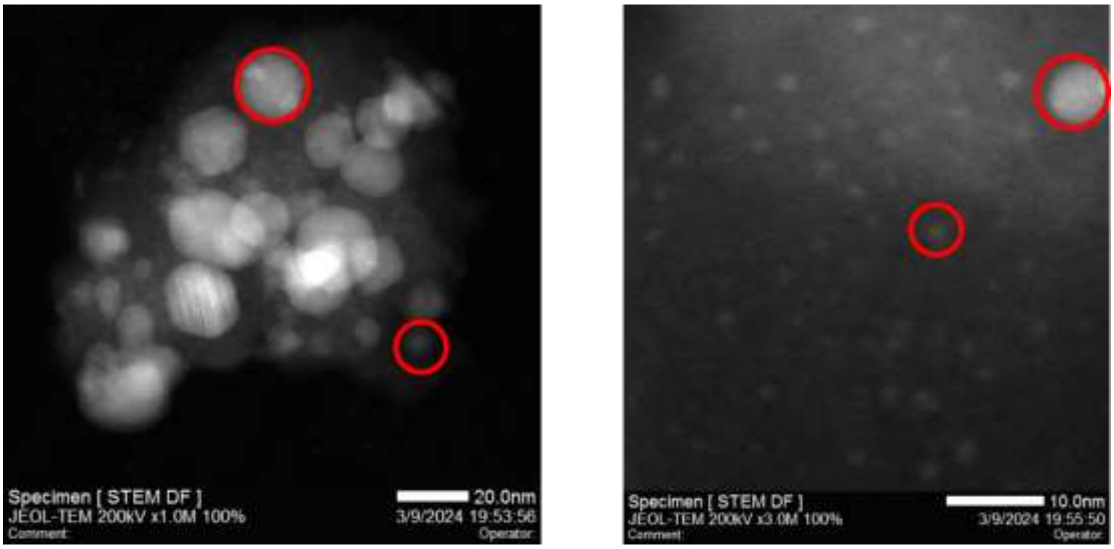


**Figure S1.** STEM images of $Co/SiO_2$ at different magnifications.

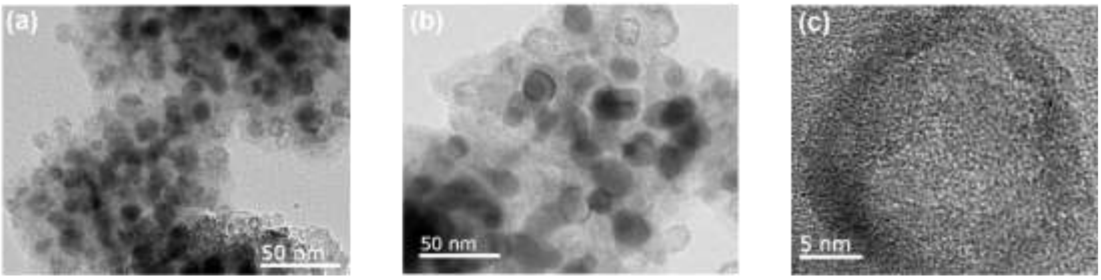


**Figure S2.** (a-c) TEM images of pre-activated $Co/SiO_2$-$H_2$.

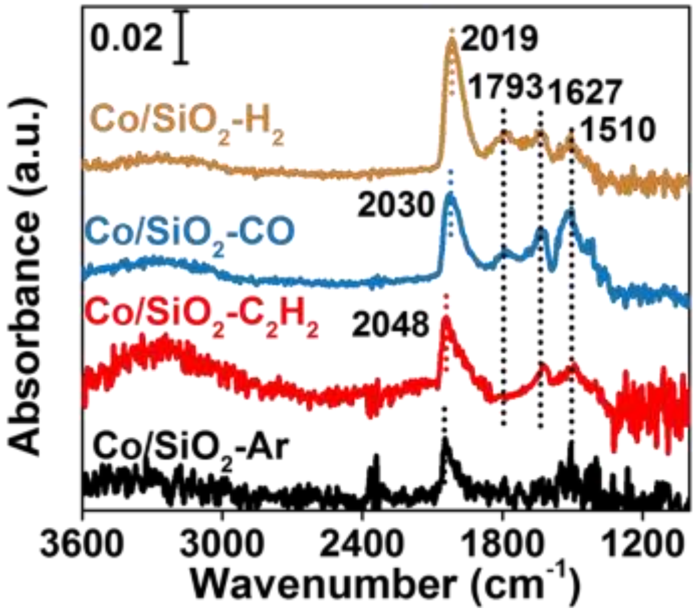


**Figure S3.** DRIFTs spectra of CO adsorption on different samples.

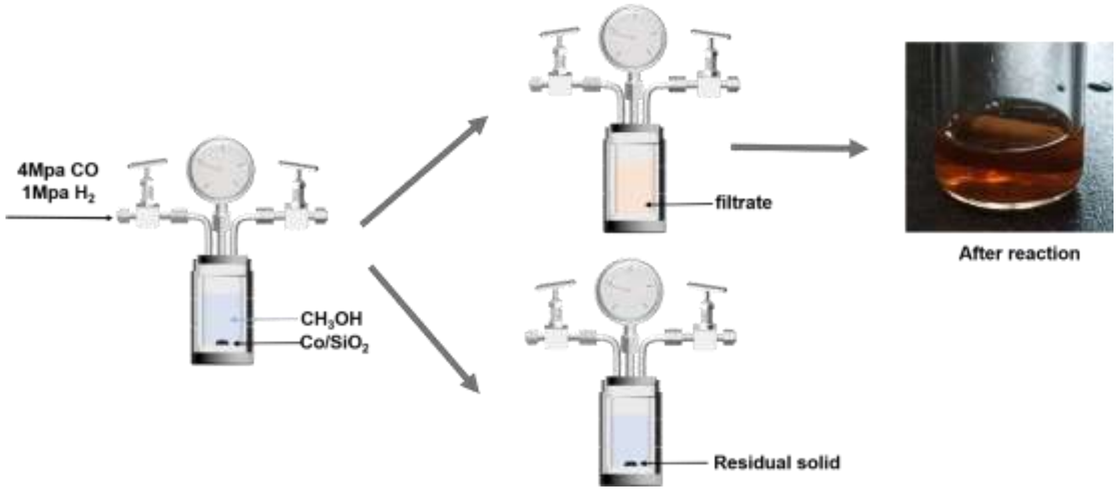


**Figure S4.** Diagram illustration of separation process.
